# GABA and Endocannabinoids Mediate Depotentiation of Schaffer Collateral Synapses Induced by Stimulation of Temperoammonic Inputs

**DOI:** 10.1371/journal.pone.0149034

**Published:** 2016-02-10

**Authors:** Yukitoshi Izumi, Charles F. Zorumski

**Affiliations:** 1 Department of Psychiatry, Washington University School of Medicine, St. Louis, MO, United States of America; 2 Taylor Family Institute for Innovative Psychiatric Research, Washington University School of Medicine, St. Louis, MO, United States of America; 3 Washington University Center for Brain Research in Mood Disorders, Washington University School of Medicine, St. Louis, MO, United States of America; Institute for Interdisciplinary Neuroscience, FRANCE

## Abstract

Long-term potentiation (LTP) of Schaffer collateral (SC) synapses in the hippocampus is thought to play a key role in episodic memory formation. Because the hippocampus is a shorter-term, limited capacity storage system, repeated bouts of learning and synaptic plasticity require that SC synapses reset to baseline at some point following LTP. We previously showed that repeated low frequency activation of temperoammonic (TA) inputs to the CA1 region depotentiates SC LTP without persistently altering basal transmission. This heterosynaptic depotentiation involves adenosine A1 receptors but not N-methyl-D-aspartate receptors, metabotropic glutamate receptors or L-type calcium channels. In the present study, we used rat hippocampal slices to explore other messengers contributing to TA-induced SC depotentiation, and provide evidence for the involvement of cannabinoid-1 and γ-aminobutyric acid (GABA) type-A receptors as more proximal signaling events leading to synaptic resetting, with A1 receptor activation serving as a downstream event. Surprisingly, we found that TA-induced SC depotentiation is independent of α-amino-3-hydroxy-5-methyl-4-isoxazolepropionic acid (AMPA)/kainate glutamate receptors. We also examined the involvement of mitogen-activated protein kinases (MAPKs), and found a role for extracellular-signal related kinase 1/2 and p38 MAPK, but not c-Jun-N-terminal kinase. These results indicate that low frequency stimulation of TA inputs to CA1 activates a complex signaling network that instructs SC synaptic resetting. The involvement of GABA and endocannabinoids suggest mechanisms that could contribute to cognitive dysfunction associated with substance abuse and neuropsychiatric disorders.

## Introduction

Defects in learning and memory accompany neuropsychiatric disorders and are a leading cause of illness-related disability. While mechanisms underlying memory are not completely understood, present evidence indicates a role for long-term, use-dependent synaptic plasticity, including long-term potentiation (LTP) and long-term depression (LTD) [1]. LTP and LTD have been extensively studied in the hippocampus, a brain region that processes new declarative memories and is involved in psychiatric illnesses.

While much has been learned about LTP and LTD [2], numerous questions remain. Among these are how hippocampal synapses reset to baseline following LTP. Is synaptic resetting a local process or can inputs from other brain regions instruct depotentiation? Because the hippocampus is involved in initial memory formation, operates over a restricted range of synaptic efficacy, and has limited storage capacity, this is an important question for understanding the dysfunction of neuropsychiatric illnesses. There are at least three ways that synaptic resetting can occur. These include homeostatic changes in which neurons adjust in response to longer-lived changes in activity by cell autonomous mechanisms [3]. Alternatively, other neurons can instruct synaptic resetting. These include homosynaptic depotentiation (LTP-D), in which the same inputs that undergo LTP trigger resetting [4,5], or heterosynaptic depotentiation in which other inputs drive resetting [6]. Considerable information is available about mechanisms underlying homeostatic [3] and homosynaptic effects [7], but less is known about heterosynaptic LTP-D. Studies to date indicate a role for N-methyl-D-aspartate receptors (NMDARs) in homosynaptic LTP-D, and this form of synaptic resetting shares some, but not all, mechanisms with *de novo* homosynaptic LTD. For example, homosynaptic LTP-D involves serine phosphatases, but differs from LTD in the role of specific subtypes of mitogen-activated protein kinases (MAPKs) [8,9,10].

Our laboratory has examined signals that induce depotentiation in the Schaffer collateral (SC) pathway and that modulate subsequent LTP in these same SC inputs [11,12]. Consistent with prior studies [4,5], we find that low frequency stimulation (LFS) of the homosynaptic SC inputs that have undergone LTP result in pathway-specific LTP-D [13]. Additionally, we found that LFS of heterosynaptic inputs that enter the CA1 region via the perforant (temperoammonic, TA) path to synapse on distal dendrites of CA1 pyramidal neurons in *stratum lacunosum moleculare* (SLM) can selectively erase SC LTP without persistently altering baseline SC transmission or subsequent SC LTP induction [11]. This latter form of LTP-D has unique properties and does not involve NMDARs, metabotropic glutamate receptors (mGluRs) or L-type voltage-activated calcium channels (VACCS), but does involve adenosine A1 receptors [11]. These latter findings indicate that activation of a heterosynaptic input to the CA1 area from entorhinal cortex depotentiates SC LTP in a manner that allows these synapses to be readily re-potentiated by subsequent homosynaptic high-frequency stimulation. Given the limited storage capacity of the hippocampus this form of depotentiation provides a mechanism by which the cortex can prepare the hippocampus for subsequent synaptic processing and avoid synaptic overload by resetting synaptic transmission in the hippocampus.

Here, we extend our work on TA-induced LTP-D by examining other messengers thought to be important in synaptic resetting, focusing on γ-aminobutyric acid (GABA) and lipid derived messengers including GABAergic neurosteroids and endocannabinoids. The involvement of GABA and endocannabinoids shown in this study indicates that low frequency stimulation of TA inputs to CA1 activates a complex signaling network that underlies this form of SC synaptic resetting.

## Results

**Fig 1:**
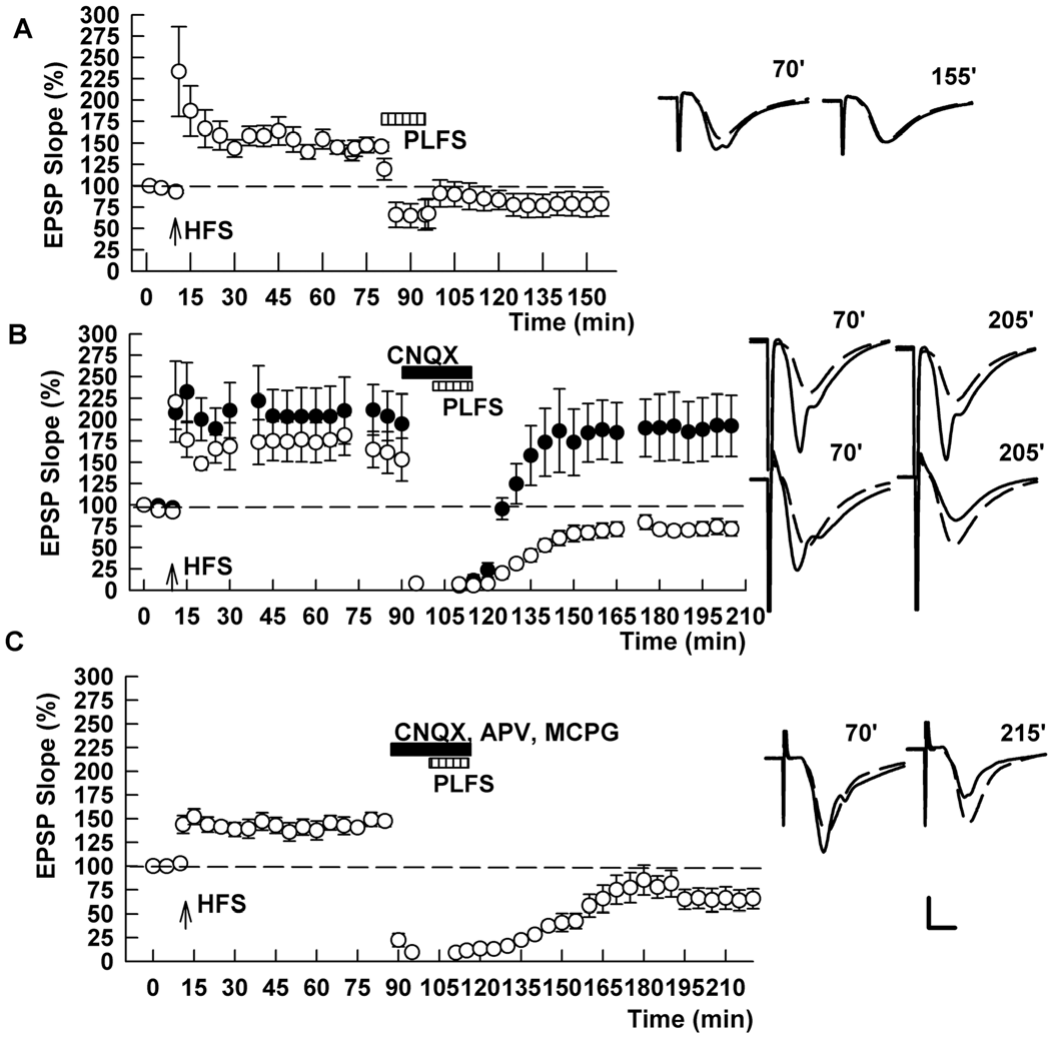
Glutamate receptor antagonists do not block TA-induced SC depotentiation. (A) The graph shows the time course of change in SC EPSPs following a single 100 Hz x 1 sec HFS delivered to the SC pathway (arrow). After establishing SC LTP, 1 Hz LFS of the TA (perforant path) inputs to CA1 (PLFS, hatched bar) reversed LTP. (B) Following stable SC LTP induction 30 μM CNQX (black bar) was administered and inhibited SC EPSPs completely. CNQX did not prevent LTP-D by PLFS (white circles) and had no effect on LTP by itself (black circles). (C) A combination of glutamate receptor antagonists (30 μM CNQX, 100 μM APV and 500 μM MCPG) also failed to prevent PLFS-induced LTP-D. Traces to the right of the graphs show representative field EPSPs at the times indicated (black lines) with baseline EPSPs shown as dashed traces. Calibration: 1 mV, 5 ms.

For the present studies, we used hippocampal slices containing a portion of entorhinal cortex to ensure intact TA inputs to area CA1. In control slices, 1 Hz x 900 pulse LFS of the TA (perforant path) inputs (referred to as PLFS in all figures) reliably reverses previously established LTP in the SC pathway, when administered 60 min following induction of SC LTP by a single 100 Hz x 1 s high frequency stimulation (HFS) of SC inputs [11] (EPSP slope: 151.2 ± 6.6% 60 min after HFS and 78.0 ± 12.1% 60 min after PLFS, n = 5, P = 0.005 by paired *t*-test after PLFS; Fig 1A). In initial experiments, we sought to determine upstream signals involved in TA-induced LTP-D. Because our prior studies found no role for NMDARs or mGluRs [11], and glutamate is the principal neurotransmitter in the TA path from entorhinal cortex to SLM [14,15], we examined the effects of inhibiting α-amino-3-hydroxy-5-methyl-4-isoxazolepropionic acid (AMPA)-type glutamate receptors. Unexpectedly, 6-cyano-7-nitroquinoxaline-2,3-dione (CNQX, 30 μM) administered during TA stimulation had no effect on depotentiation, despite inhibiting synaptic responses in the SC pathway completely during perfusion (145.0 ± 10.4% 60 min after HFS and 89.2 ± 15.1% 60 min after PLFS, n = 5, P = 0.005 by paired *t*-test after PLFS; Fig 1B). The depotentiation induced by TA LFS in the presence of CNQX did not simply result from the complete block of postsynaptic responses, because administering CNQX in the absence of TA stimulation had no effect on LTP following washout of CNQX (144.1 ± 9.2% 60 min after HFS and 146.9 ± 21.2% after washout, n = 5, p = 0.91; Fig 1B). We also examined the effect of combining glutamate receptor antagonists, but found that a combination of 30 μM CNQX, 100 μM d,l-2-amino-5-phosphonovalerate (APV) and 500 μM α-methyl-4-carboxyphenylglycine (MCPG) to block AMPA/kainate, NMDA and mGluR receptors did not prevent TA-induced LTP-D (143.0 ± 9.5% 60 min after HFS and 62.2 ± 10.2% 60 min after PLFS, N = 5; Fig 1C).

**Fig 2:**
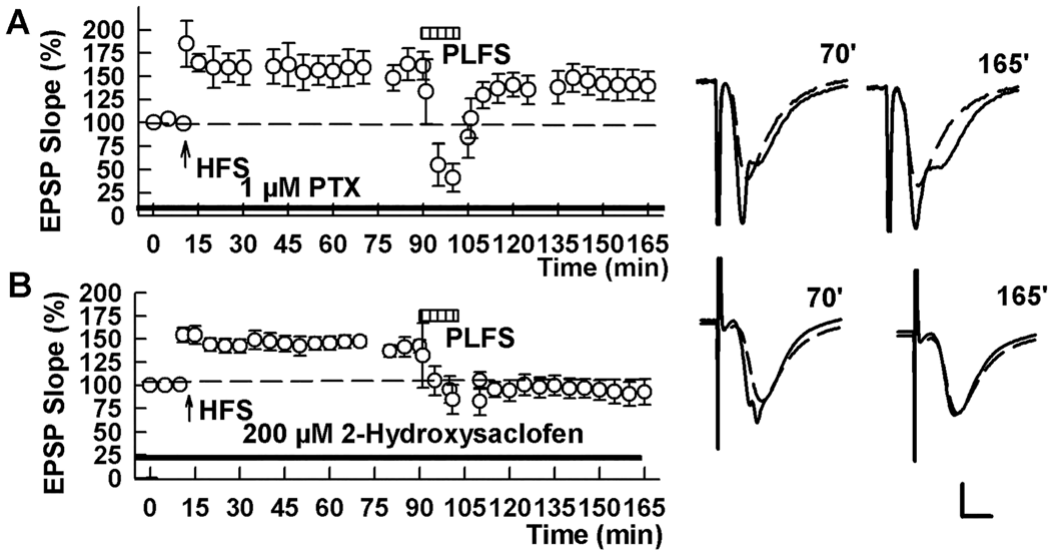
GABA-A but not GABA-B receptors contribute to TA-induced SC LTP-D. (A) In the presence of 1 μM picrotoxin, a GABA-A receptor antagonist (PTX, black bar), PLFS (hatched bar) failed to depotentiate SC LTP. (B) 2-Hydroxysaclofen (200 μM, black bar), a GABA-B receptor antagonist did not alter TA-induced LTP-D. Traces show representative EPSPs at the times indicted in the graphs. Calibration: 1 mV, 5 ms.

Inputs from entorhinal cortex to CA1 SLM via the TA pathway are topographically precise and activate a small number of CA1 pyramidal neurons [16,17]. SLM inputs, however, also activate a more widely distributed feedforward GABAergic inhibitory system [18,19]. These observations prompted us to examine whether block of GABA-A receptors alters TA-induced LTP-D. We found that 1 μM picrotoxin (PTX), a non-competitive and broad spectrum GABA-A receptor antagonist, completely prevented TA LTP-D when administered prior to and during TA LFS (148.2 ± 11.8% 60 min after HFS and 135.6 ± 7.4% 60 min after PLFS, n = 5, P = 0.28 before and after PLFS; Fig 2A). In contrast, the GABA-B receptor antagonist 2-hydroxysaclofen (200 μM) had no effect on TA-induced LTP-D (142.4 ± 7.0% 60 min after HFS and 78.8 ± 10.7% 60 min after PLFS, n = 5, P = 0.004; Fig 2B).

**Fig 3:**
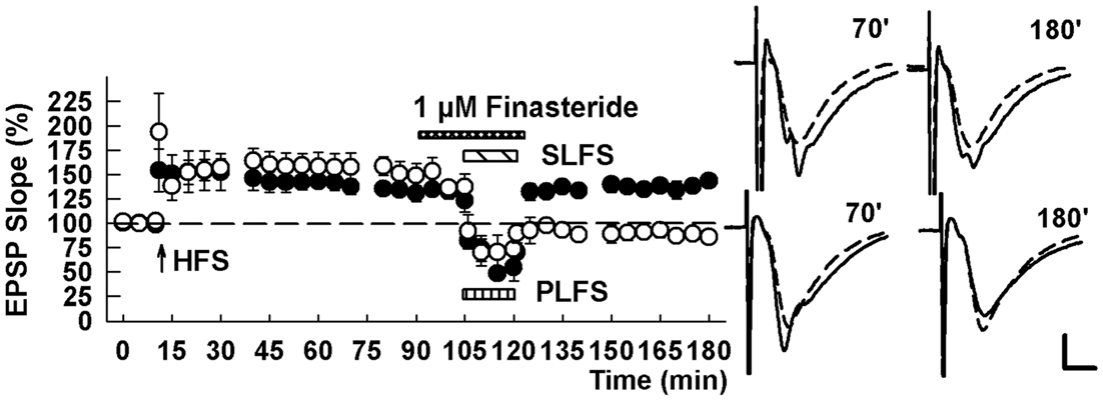
GABAergic neurosteroids contribute to homosynaptic LTP-D in the SC pathway but not to TA-induced LTP-D. The graph shows the ability of 1 μM finasteride, a 5α reductase inhibitor that blocks synthesis of 5α-reduced neurosteroids, to block LTP-D induced by 1 Hz stimulation of the homosynaptic SC pathway (black circles). In contrast, finasteride had no effect on SC LTP-D induced by PLFS (hatched bar) in a separate set of slices (white circles). HFS was administered to the SC pathway at the arrow. Traces show representative EPSPs at the times indicated. Calibration bar: 1 mV, 5 ms.

GABA-A receptors can be positively modulated and directly activated by 5α-reduced neurosteroids, including allopregnanolone [20], and our prior studies indicated a role for GABA and GABA-enhancing neurosteroids in *de novo* homosynaptic LTD in the SC pathway [21]. These observations prompted us to examine a role for GABAergic neurosteroids in TA-induced LTP-D using the 5α-reductase inhibitor, finasteride (1 μM), to block the synthesis of allopregnanolone and other 5α-reduced neurosteroids. Finasteride had no effect on TA-induced LTP-D, indicating that neurosteroid production does not participate in this form of synaptic modulation (139.4 ± 6.3% 60 min after HFS and 76.3 ± 8.3% after PLFS, n = 5, P = 0.001; Fig 3). In contrast, finasteride completely inhibited homosynaptic LTP-D (137.7 ± 7.7% 60 min after HFS and 143.7 ± 4.0% 60 after Schaffer LFS, n = 5, P = 0.599; Fig 3), consistent with what we previously showed for *de novo* SC LTD [21].

**Fig 4:**
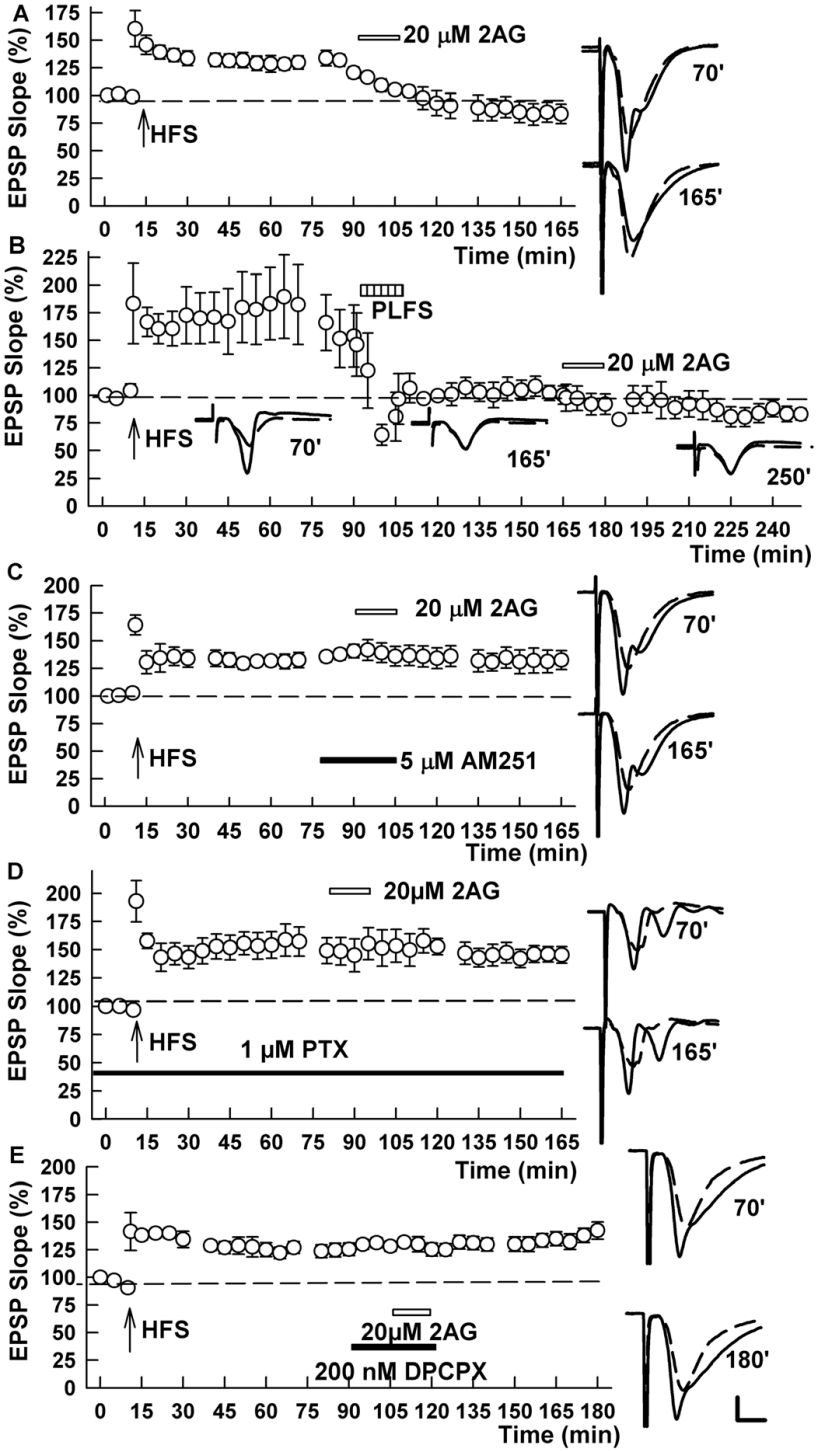
Exogenous administration of an endocannabinoid produces chemical depotentiation of SC LTP. (A) Perfusion of 20 μM 2AG (white bar), an endocannabinoid agonist, reversed LTP established by SC HFS (arrow). (B) Following LTP-D induced by PLFS, 20 μM 2AG failed to produce further suppression of SC EPSPs. (C) The effects of 2AG (white bar) on SC LTP were blocked by 5 μM AM251 (black bar), an inhibitor of CB1 receptors. (D,E) Similarly, the effects of 2AG on SC LTP were blocked by pretreatment with 1 μM PTX (D) and 200 nM DPCPX, an adenosine A1 receptor antagonist (E). Traces show representative EPSPs at the times indicated in the graphs. Calibration: 1 mV, 5 ms.

Based on prior studies demonstrating a role for endocannabinoids (ECs) in CA1 synaptic depression and LTD [22,23,24], we also examined whether ECs contribute to TA-induced LTP-D. When administered for 15 minutes following stable induction of SC LTP, exogenous administration of the EC, 2-arachidonylglycerol (2-AG, 20 μM), resulted in chemically-induced depotentiation (122.6 ± 2.1% after HFS and 82.7 ± 15.5% after 2AG, n = 5, P = 0.003; Fig 4A). We also found that LTP-D induced by TA LFS occluded further depression by 2AG, suggesting commonality of action (163.9 ± 24.5% 60 min after SC HFS, 87.8 ± 3.7% 60 min following TA LFS, and 90.0 ± 4.7% 60 min following 2AG, n = 5, P = 0.435 vs. TA LFS; Fig 4B). The effects of 2-AG, were not mimicked by a related EC, anandamide (145.0 ± 6.4% 60 min after LFS and 146.7 ± 5.2% 60 min after 10 μM anandamide, n = 3, P = 0.851), but were completely inhibited by the CB1 receptor antagonist, AM251 (133.7 ± 5.4% after HFS and 136.5 ± 11.1% 60 min after 2AG, n = 5, P = 0.714; Fig 4C). Interestingly, the depotentiating actions of 2-AG were also completely blocked by PTX (163.0 ± 19.6% after HFS and 145.2 ± 14.4% after 2AG, n = 6, P = 0.467; Fig 4D). Depotentiation by 2-AG was also inhibited by the adenosine A1 receptor antagonist, 8-cyclopenthyl-1,3-dipropylxanthine (DPCPX) (200 nM) (139.1 ± 8.3% after HFS and 141.6 ± 10.9% after 2AG, n = 6, P = 0.724; Fig 4E), which we previously found to block TA-induced LTP-D [11].

**Fig 5:**
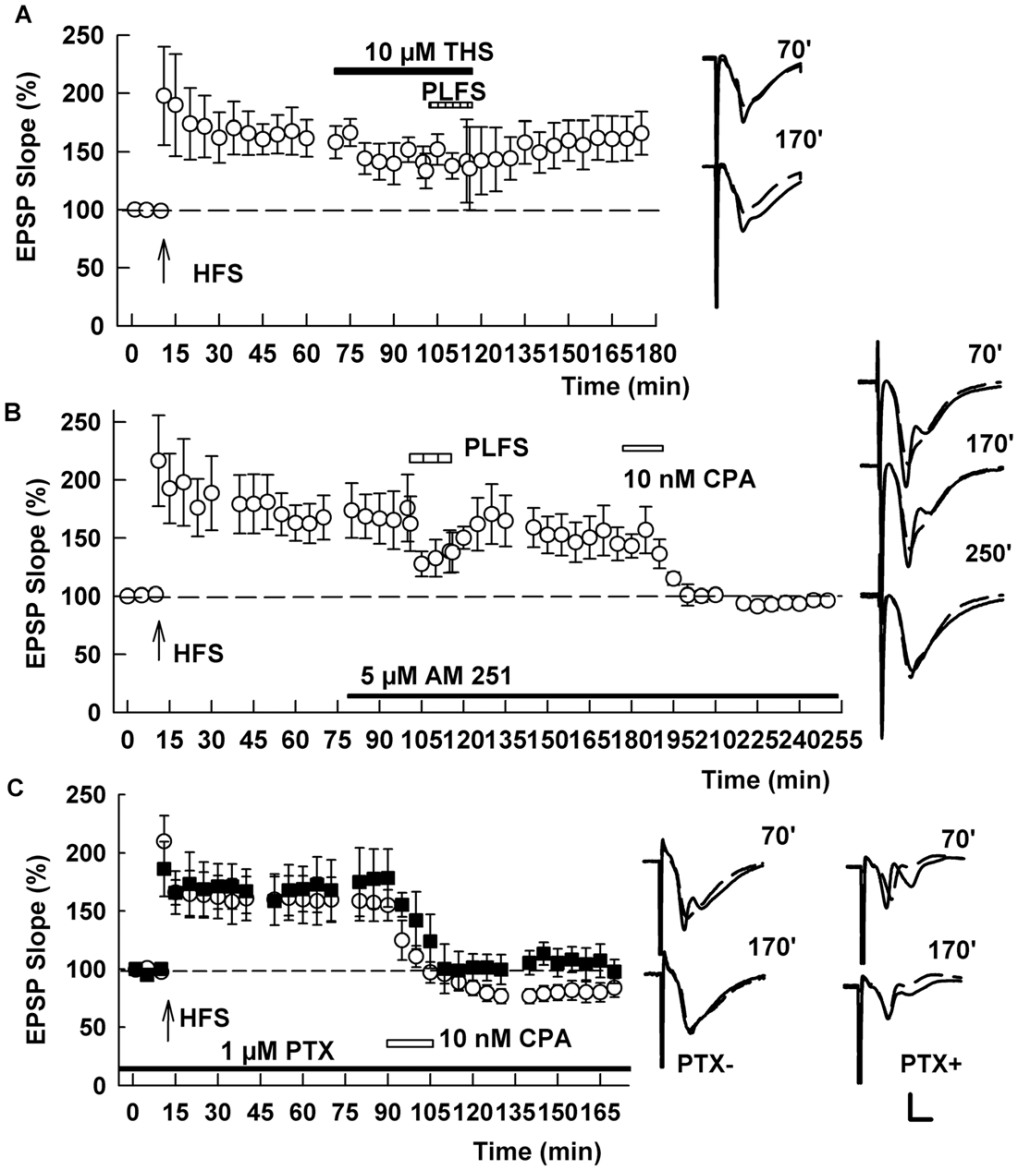
Endocannabinoids contribute to TA-induced LTP-D of SC synapses. (A) Treatment of slices with 10 μM tetrahydrolipstatin (THS, black bar), an inhibitor of diacylglycerol lipase and 2AG synthesis, blocked the ability of PLFS (hatched bar) to depotentiate SC LTP. SC HFS was administered at the arrow. (B) TA-induced LTP-D was also blocked by 5 μM AM251, a CB1 receptor antagonist (black bar). AM251, however, did not block the ability of 10 nM cyclopentyladenosine (CPA, white bar), an adenosine A1 receptor agonist, to induce chemical depotentiation of SC LTP. (C) PTX (black bar) also failed to alter depotentiation by CPA (white bar) as shown with black squares. White circles show effects of CPA in the absence of PTX. Traces show representative EPSPs. Calibration: 1 mV, 5 ms.

Further supporting a role for endogenous ECs in TA LTP-D, we found that tetrahydrolipstatin (10 μM), an inhibitor of diacylglycerol lipase that blocks endogenous 2-AG synthesis [25], prevented the effects of TA LFS on established SC LTP (169.5 ± 13.8% after HFS and 149.7 ± 12.7% after PLFS, n = 5, p = 1.000 by signed Rank test; Fig 5A). We also found that AM251, the CB1 receptor antagonist, blocked the effects of TA LFS on LTP, but did not prevent the ability of the adenosine A1 receptor agonist, N^6^-cyclopentyl-adenosine (CPA, 10 nM) to reverse previously established SC LTP (136.8 ± 7.6% after HFS and 134.0 ± 5.3% after PLFS and 84.5 ± 6.3% after CPA, n = 6, p = 0.011; Fig 5B). Similarly, the ability of CPA to depotentiate SC synapses was not altered by PTX (172.0 ± 26.6% after HFS and 100.5 ± 15.8% after PLFS, n = 5, p = 0.023; Fig 5C). Taken together these results indicate that both ECs and adenosine play a role in TA-induced LTP-D, but based on specific receptor antagonists, adenosine A1 receptor activation appears to occur downstream of CB1 receptor activation. Furthermore, activation of GABA_A_ receptors appears to occur downstream of ECs but upstream of adenosine receptors.

**Fig 6:**
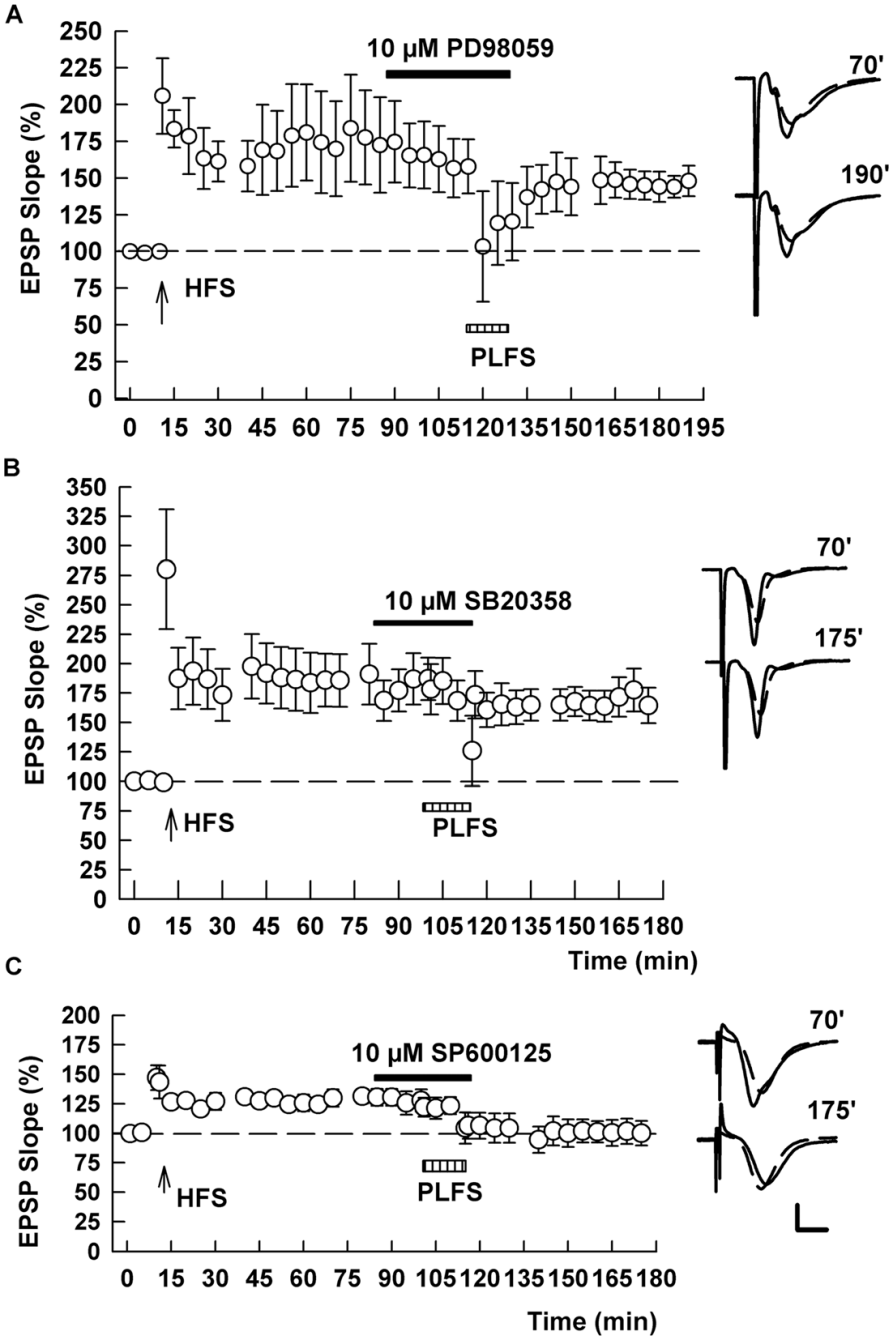
The MAP kinases ERK 1/2 and p38 contribute to TA-induced LTP-D. (A) In the presence of 10 μM PD98059 (black bar), a MEK inhibitor that blocks signaling in the ERK pathway, PLFS (hatched bar) failed to induce LTP-D. HFS of the SC pathway was administered at the arrow. (B) Similarly, administration of 10 μM SB20358, a p38 MAPK inhibitor, also blocked TA-induced LTP-D. (C) In contrast, 10 μM SP600125, an inhibitor of the JNK pathway, did not prevent PLFS-induced LTP-D. Traces show representative EPSPs at the times indicated. Calibration: 1 mV, 5 ms.

Because prior studies indicate that different MAPKs participate in hippocampal LTD and depotentiation [10], we examined a role for MAPKs in TA-induced LTP-D. We found that the MEK inhibitor, PD98059 (10 μM), which prevents signaling in the ERK 1/2 pathway (159.5 ± 15.6% after HFS and 147.2 ± 15.7% after PLFS, n = 8, Fig 6A), and the p38 MAPK inhibitor, SB20358 (10 μM) (160.5 ± 16.5% after HFS and 155.3 ± 12.2% after PLFS, n = 5, Fig 6B) [11], independently blocked the effects of TA stimulation on LTP, suggesting a role for these MAPKs in heterosynaptic depotentiation. In contrast, the c-Jun-N-terminal kinase (JNK) inhibitor, SP600125 (10 μM) had no effect on TA-induced LTP-D (138.4 ± 8.8% after HFS and 91.4 ± 12.5% after PLFS, n = 5, p = 0.008; Fig 6C).

**Fig 7:**
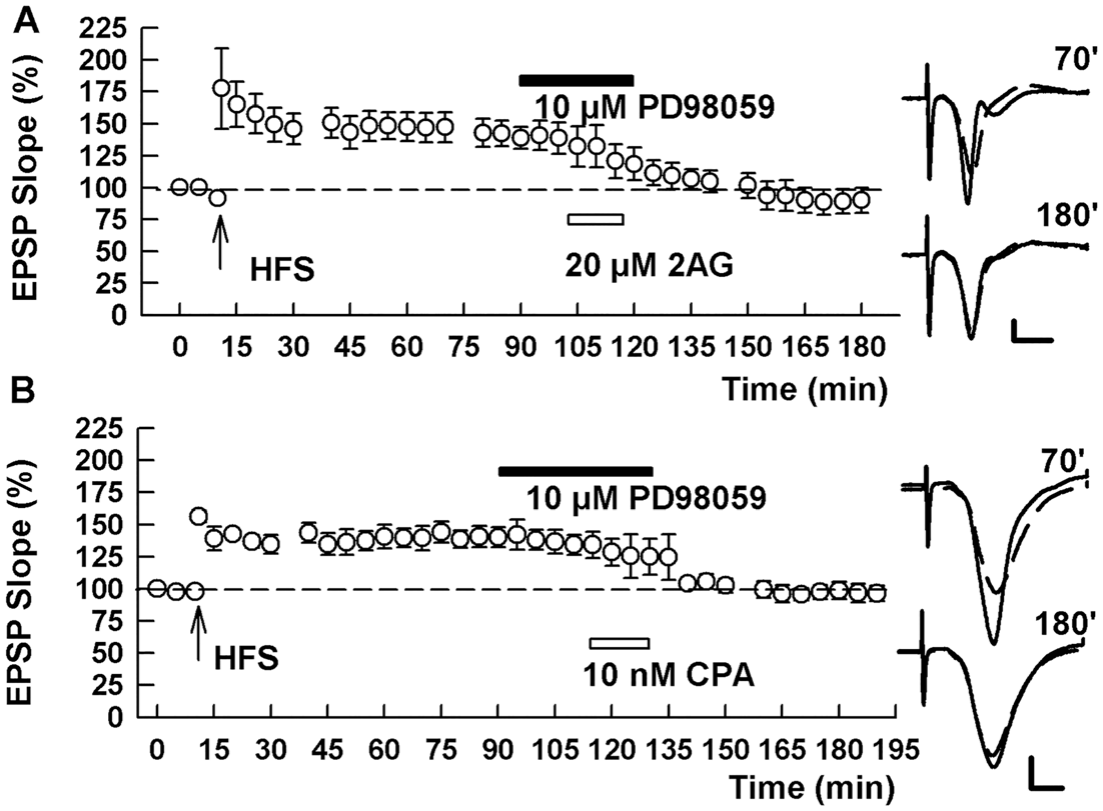
An ERK inhibitor does not alter chemical LTP-D by 2AG or CPA. (A,B) In the presence of 10 μM PD98059, both 2AG (A), the endocannabinoid agonist, and CPA (B), the adenosine A1 receptor agonist, induced chemical depotentiation. Traces show EPSPs at the times indicated. Calibration: 1 mV, 5 ms.

**Fig 8:**
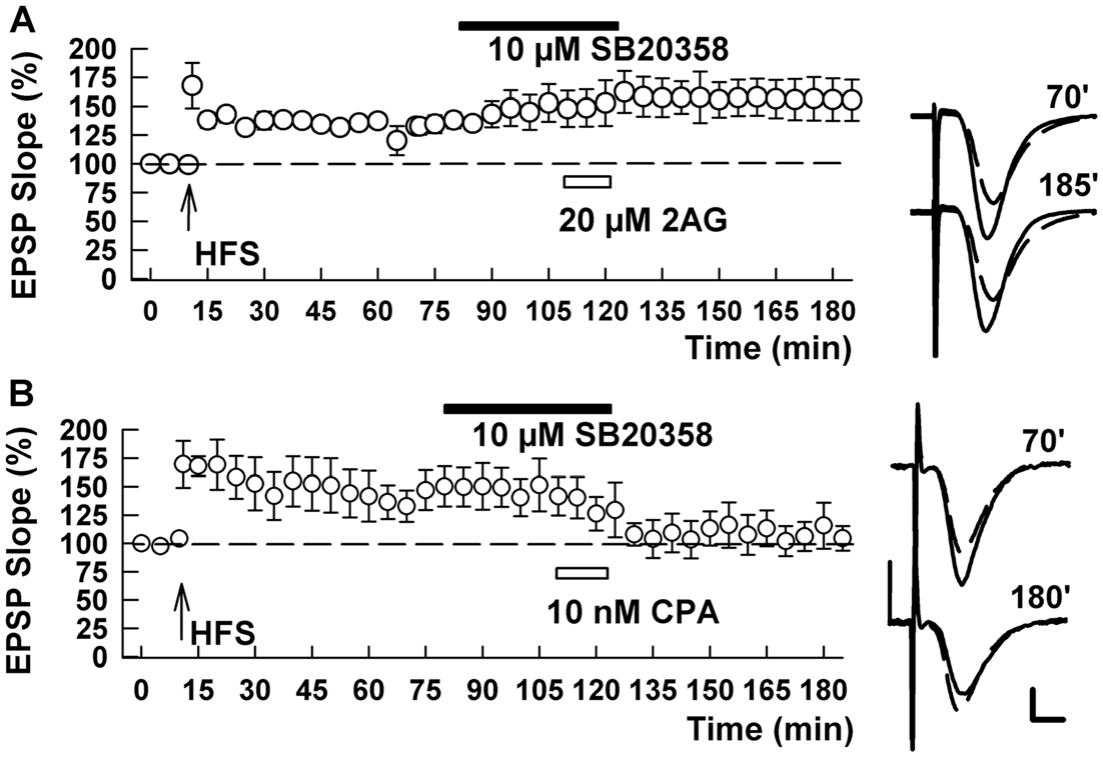
A p38 MAPK inhibitor blocks chemical LTP-D by CB1 receptor activation but not A1 receptor activation. (A) In the presence of 10 μM SB20358, 20 μM 2AG failed to induce LTP-D of SC LTP. B. SB20358, however, did not prevent chemical depotentiation by CPA. Traces show EPSPs at the times indicated. Calibration 1 mV, 5 ms.

Finally, we examined whether the MAPK inhibitors altered chemically-induced depotentiation using the receptor agonists described above. We found the PD98059, failed to block the effects of either 2-AG (137.0 ± 11.5% after HFS and 83.9 ± 7.9% after 2AG, n = 5, p = 0.002; Fig 7A) or CPA (140.0 ± 5.8% after HFS and 92.3 ± 8.5% after CPA, n = 6, p = 0.004; Fig 7B) on LTP, suggesting that ERK activation occurs relatively early in the depotentiation cascade and is upstream of both CB1 and A1 receptor activation. In contrast, the p38 MAPK inhibitor, SB20358 completely eliminated LTP-D produced by 2-AG (134.4 ± 2.8% after HFS and 151.5 ± 12.9% after 2AG, n = 5, p = 0.216; Fig 8A), but did not prevent chemical depotentiation induced by the adenosine agonist (157.8 ± 23.1% after HFS and 99.4 ± 12.1% after CPA, n = 6, p = 0.0239; Fig 8B).

## Discussion

**Fig 9:**
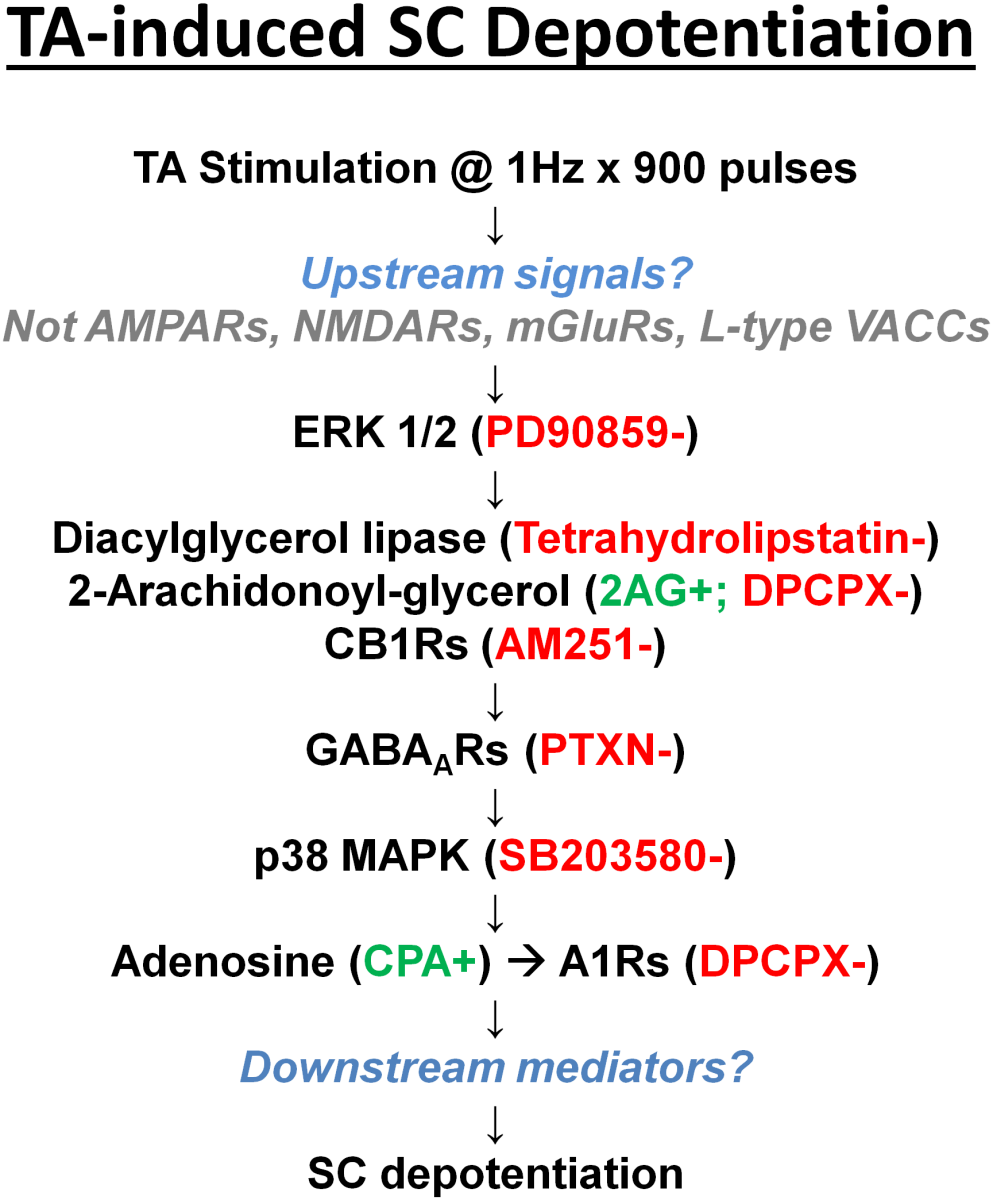
Mechanisms contributing to TA-induced depotentiation. The figure presents a scheme for signaling involved in TA-induced SC LTP-D based on the effects of selective agonists and antagonists. Based on results to date, activation of ERK 1/2 appears to occur early in the cascade, while A1R activation is a late event.

These results indicate that low frequency stimulation of inputs to distal dendrites in the SLM region of hippocampal area CA1 depotentiates previously established SC LTP via a complex signaling system. We previously found that this form of heterosynaptic depotentiation in which extrahippocampal inputs instruct synaptic resetting of SC synapses involves activation of adenosine A1 receptors but not NMDARs, mGluRs or L-type calcium channels [11]. Our present study indicates that TA LTP-D engages multiple other modulators, involving at the minimum activation of CB1 receptors, GABA-A receptors and A1 receptors, along with two components of MAPK signaling, ERK 1/2 and p38 MAPK. Based on differential effects of inhibitors of these modulators and pathways, it appears that endocannabinoids and CB1 receptors are early mediators, GABA and GABA-A receptors are involved at an intermediate stage, and A1 receptor activation occurs more distally in the cascade. Similarly, ERK 1/2 is involved more proximally, while p38 MAPK appears to play a role more distally, but prior to A1 receptor involvement (Fig 9).

A surprising result is the apparent lack of involvement of glutamate receptors in this form of depotentiation. We previously found no role for NMDARs or mGluRs based on broad spectrum antagonists [11]. Here we examined a role for AMPA/kainate receptors using CNQX, a broad spectrum antagonist that blocked SC transmission completely and reversibly at the concentration used. Despite the block of SC EPSPs, CNQX had no effect on the ability of TA LFS to depotentiate SC LTP. Similarly a combination of antagonists against AMPA/kainate, NMDA and mGluR receptors failed to prevent TA-induced SC depotentiation. We used MCPG as a broad spectrum mGluR antagonist in these studies, although this agent has diminished effects at Group III mGluRs [26]).

The findings with glutamate receptor antagonists suggest that other modulators entering the CA1 region via the TA path are critical activators of this unique form of LTP-D. Activation of ECs and GABAergic inputs is likely based on the effects of AM251 and picrotoxin, and the known ability of SLM inputs to engage feed forward inhibition [18]. For experiments examining the role of GABA-A receptors, we used the broad spectrum antagonist, picrotoxin, dissolved in ethanol and administered at 1 μM. We previously found that this concentration of picrotoxin dampens CA1 inhibition without inducing epileptiform activity. At this low concentration, picrotoxin may preferentially block liganded extrasynaptic GABA-A receptors although our studies do not directly address this issue [27]. Furthermore, recent studies indicate that entorhinal cortex sends direct long-range GABAergic inhibitory projections to CA1 via SLM and these inputs can modulate CA1 function [28]. Other modulators, including monoamines, also innervate SLM and could play a role [29,30,31]. Our studies exclude a role for GABA-enhancing, 5α-reduced neurosteroids based on the lack of effect of finasteride, a selective 5α-reductase inhibitor that blocks synthesis of allopregnanolone in the hippocampus [20, 32,33]. These neurosteroids, however, do play a role in homosynaptic SC LTP-D, just as we previously found for homosynaptic SC LTD [21].

Our results indicate that ECs, particularly 2AG, and CB1 receptors are involved in TA-induced depotentiation. Prior studies have shown that ECs play complex roles in synaptic function and plasticity, participating in homosynaptic and heterosynaptic LTD in the SC pathway [34,35]. In some cases, the involvement of ECs has included a complex interaction with NMDARs and mGluRs, particularly Group I mGluRs linked to phosphoinositide metabolism [24,34,36]. ECs also modulate GABA-mediated inhibitory transmission, including forms of inhibitory LTD (I-LTD) [37]. Thus, interactions of ECs with interneurons and GABA-A receptor activation as observed here are consistent with findings in other forms of plasticity, including our own prior studies of homosynaptic SC LTD [24]. ECs are known to function as retrograde intercellular messengers in synaptic plasticity [22,23] raising the possibility that these modulators help to drive both presynaptic and postsynaptic changes in TA-induced LTP-D. In our studies, it appears that ECs are involved relatively early in the events leading to synaptic resetting.

The present studies also begin to explore second messenger systems contributing to TA-induced LTP-D. Prior work has indicated that MAPKs play important role in several forms of long-term synaptic plasticity, including LTP, LTD and LTP-D [10,38,39]. Here we found that ERK 1/2 and p38 MAPK, but not JNK, are involved in TA-induced LTP-D. ERK 1/2 appears to be involved earlier in the process and p38 MAPK later, based on block by specific antagonists and the effects of these MAPK antagonists on agonists that activate CB1 and A1 receptors. Prior studies indicate that p38 MAPK is involved in SC LTD [39,40,41] and in NMDAR-mediated metaplastic inhibition of SC LTP [42]. It is likely that other intracellular and possibly intercellular messengers are involved in TA-induced LTP-D, based on the role that nitric oxide [13] and phosphatases [2] play in homosynaptic SC LTD and LTP-D. It is also likely that TA-induced LTP-D modulates the trafficking of AMPARs at SC synapses based on the importance of this mechanism in multiple forms of hippocampal synaptic plasticity [39,43].

Our studies further indicate that adenosine acting at A1 receptors is involved later in the events driving TA-induced LTP-D than other messengers examined to date. Among the signaling inhibitors studied, only the A1 receptor antagonist blocked the effects of an adenosine agonist, and this antagonist also blocks the effects of direct TA stimulation [11]. The involvement of adenosine in this form of LTP-D is consistent with its role in other forms of LTD and depotentiation [44,45,46,47]. Numerous questions remain about the involvement of adenosine in TA stimulation, including the source of adenosine and the messengers that adenosine activates to ultimately produce depotentiation. Prior work indicates that both neurons and glia can release adenosine and, in some cases, adenosine is generated from adenosine triphosphate (ATP) followed by enzymatic conversion by extracellular ectonucleotidases [41,48]. It is also unclear which messengers/mechanisms are triggered by adenosine to drive synaptic resetting. We examined a role for p38 MAPK based on prior work indicating that adenosine can activate p38 MAPK to depotentiate synapses [47], but found that a p38 inhibitor did not alter the effects of an A1 receptor agonist, suggesting that p38 activation likely occurs earlier in the signaling events.

Taken together, the present results indicate that TA-induced LTP-D is a unique form of synaptic plasticity and provides a mechanism by which extra-hippocampal brain regions can instruct the resetting of SC synapses following LTP induction. Prior work has indicated the importance of bidirectional synaptic plasticity and metaplasticity in learning [2,12] and TA inputs are subject to both LTP and LTD [49,50,51] and can modulate induction of SC plasticity and learning [52,53,54,55]. The complex signaling involved in TA-induced LTP-D, including the role of GABA, ECs and adenosine, also provide ways by which therapeutic and abused drugs can modulate hippocampal function. Understanding this form of synaptic resetting could provide ways by which cognitive function can be improved in a variety of neuropsychiatric disorders.

## Materials and Methods

### Hippocampal Slice Preparation

Hippocampal slices were prepared from postnatal day (P) 30–32 albino rats using standard methods [11,29]. Rats were anesthetized with isoflurane and decapitated. All animal protocols were approved by the Washington University Animal Studies Committee in accordance with national and international guidelines. Dissected hippocampi were placed in ice-cold artificial cerebrospinal fluid (ACSF) containing (in mM): 124 NaCl, 5 KCl, 2 MgSO_4_, 2 CaCl_2_, 1.25 NaH_2_PO_4_, 22 NaHCO_3_, 10 glucose, bubbled with 95% O_2_-5% CO_2_ at 4–6°C, and cut into 450 μm slices using a vibrotome. The slices were cut in a fashion that included a significant portion of entorhinal cortex to maximize TA inputs to SLM in the CA1 region [11]. Acutely prepared slices were placed in an incubation chamber containing gassed ACSF for 1 hr at 30°C before further experimentation.

### Hippocampal Slice Physiology

At the time of study, slices were transferred individually to a submersion-recording chamber. Experiments were done at 30°C with continuous ACSF perfusion at 2 ml/min. Extracellular recordings were obtained from the apical dendritic layer (*stratum radiatum*) of the CA1 region for analysis of excitatory postsynaptic potentials (EPSPs) using electrodes filled with 2 M NaCl (5–10 MΩ resistance).

EPSPs were evoked with 0.1 msec constant current pulses through a bipolar stimulating electrode in the SC pathway. A second stimulating electrode was placed in the TA pathway to activate distal dendrites of CA1 in SLM. A control input-output curve was obtained to determine stimulus intensities for subsequent studies. Responses were monitored by applying single stimuli to the SC pathway every 60 sec at half maximal intensity. After establishing a stable baseline for at least 10 min, LTP was induced by a single 100 Hz x 1 s tetanus using the same intensity stimulus. Input-output curves were repeated 60 min following tetanic stimulation. In some experiments, a second independent SC input to CA1 was activated using a stimulating electrode placed at a different level in *stratum radiatum* than the primary SC stimulating electrode and positioned on the distal (subiculum) side of the dendritic recording electrode.

### Materials

2-Arachidonylglycerol (2AG), AM251, PD98059, SB20358 and SP600125 were obtained from Tocris Bioscience (St. Louis MO). Finasteride was obtained from Steraloids (Newport RI). Other chemicals and pharmacological agents were obtained from Sigma Chemical Company (St. Louis MO).

### Statistical Analysis

Data were collected and analyzed using PClamp software (Axon Instruments, Union City CA). Data in the text are expressed as mean ± SEM. A two-tailed Student’s t-test was used for comparisons between groups. Statistical comparisons were based on input-output curves at baseline and sixty minutes following tetanic or 1 Hz stimulation with p < 0.05 considered significant. Analyses were done using commercial software (SigmaStat, Systat Software, Inc., Richmond City, CA).
